# RNA-binding protein transcripts as potential biomarkers for detecting Primary Sclerosing Cholangitis and for predicting its progression to Cholangiocarcinoma

**DOI:** 10.3389/fmolb.2024.1388294

**Published:** 2024-06-06

**Authors:** Ugo Ala, Sharmila Fagoonee

**Affiliations:** ^1^ Department of Veterinary Sciences, University of Turin, Turin, Italy; ^2^ Institute of Biostructure and Bioimaging (CNR), Molecular Biotechnology Center “Guido Tarone”, Turin, Italy

**Keywords:** Primary Sclerosing Cholangitis, cholangiocarcinoma, RNA-binding proteins, extracellular vesicles, biomarkers, data mining, bioinformatics

## Abstract

Primary Sclerosing Cholangitis (PSC) is a persistent inflammatory liver condition that affects the bile ducts and is commonly diagnosed in young individuals. Despite efforts to incorporate various clinical, biochemical and molecular parameters for diagnosing PSC, it remains challenging, and no biomarkers characteristic of the disease have been identified hitherto. PSC is linked with an uncertain prognosis, and there is a pressing need to explore multiomics databases to establish a new biomarker panel for the early detection of PSC’s gradual progression into Cholangiocarcinoma (CCA) and for the development of effective therapeutic interventions. Apart from non-coding RNAs, other components of the Ribonucleoprotein (RNP) complex, such as RNA-Binding Proteins (RBPs), also hold great promise as biomarkers due to their versatile expression in pathological conditions. In the present review, an update on the RBP transcripts that show dysregulated expression in PSC and CCA is provided. Moreover, by utilizing a bioinformatic data mining approach, we give insight into those RBP transcripts that also exhibit differential expression in liver and gall bladder, as well as in body fluids, and are promising as biomarkers for diagnosing and predicting the prognosis of PSC. Expression data were bioinformatically extracted from public repositories usingTCGA Bile Duct Cancer dataset for CCA and specific NCBI GEO datasets for both PSC and CCA; more specifically, RBPs annotations were obtained from RBP World database. Interestingly, our comprehensive analysis shows an elevated expression of the non-canonical RBPs, *FANCD2*, as well as the microtubule dynamics regulator, *ASPM*, transcripts in the body fluids of patients with PSC and CCA compared with their respective controls, with the same trend in expression being observed in gall bladder and liver cancer tissues. Consequently, the manipulation of tissue expression of RBP transcripts might be considered as a strategy to mitigate the onset of CCA in PSC patients, and warrants further experimental investigation. The analysis performed herein may be helpful in the identification of non-invasive biomarkers for the early detection of PSC and for predicting its progression into CCA. In conclusion, future clinical research should investigate in more depth the full potential of RBP transcripts as biomarkers for human pathologies.

## 1 Introduction

According to a recent report on worldwide incidence, biliary tract cancers are ascribed as rare due to the overall incidence rate ranging from 12.42 to 1.12/100,000 person-years (Baria et al., 2022). Biliary tract cancers encompass a heterogeneous group of malignant epithelial hepatic and perihepatic malignancies originating from the biliary tree. Their anatomical subtypes, including gallbladder cancer, cholangiocarcinoma (CCA) and ampulla of Vater cancer, exhibit significant variations across different geographic regions. Owing to the asymptomatic nature of the biliary tract cancers, diagnosis often occurs at advanced stages of the disease, limiting treatment options. The 5-year survival rate for patients with biliary tract cancers in the United States and Europe is less than 20% (Baria et al., 2022). CCA stands out as the most common cancer found in individuals diagnosed with Primary Sclerosing Cholangitis (PSC), with a reported lifetime occurrence reaching approximately 13% (Bergquist et al., 2002; Villard et al., 2023). The incidence of CCA in PSC patients peaks within the initial year of PSC diagnosis, followed by a yearly occurrence rate of 0.5%–1.5% (Villard et al., 2023). PSC is a chronic inflammatory liver disorder affecting the bile ducts and commonly diagnosed in young patients. The bile ducts are exposed to diverse pathological triggers including exposure to inflammatory cytokines, chronic cholestasis and infections, oxidative stress causing aberrant cellular proliferation, reduction in DNA repair and resistance to apoptosis (Catanzaro et al., 2023). All these events culminate in the development of intermittent strictures and dilatations along the ducts, accompanied by periductal fibrosis (Tornai et al., 2022). In the majority of cases, this condition advances to cirrhosis and a stage of decompensated disease. The progression rate in PSC varies widely, and accurately predicting the disease trajectory is crucial for clinical practice and designing interventional trials. Albeit attempts at incorporating multiple parameters, such as clinical, laboratory, radiological, and histological, have been made, diagnosing PSC poses challenges, and as of now, no biomarkers characteristic of the disease have been identified (Tornai et al., 2022; Mulinacci et al., 2023). The considerable disease variability between patients and oscillating liver biochemistries warrant the search for new biomarkers that can sort out subjects with high risk of developing end-stage liver disease (Fossdal et al., 2021). In the era of high throughput sequencing and digital advancements, there is need for stratified medicine in PSC (Mulinacci et al., 2023). PSC is associated with uncertain prognosis and exploring biomarker discovery-oriented multiomics databases to design a novel biomarker panel for the early detection of CCA arising from PSC and for developing effective therapeutic interventions has become a necessity.

### 1.1 RNA metabolism in human diseases

Multilayered interconnected regulatory networks ensure the correct functioning of the hepatobiliary system. Proteostasis or the dynamic regulation of the cellular proteome to maintain a balanced homeostasis is an important layer of these networks. It refers to the biological mechanisms controlling key processes such as protein biosynthesis, post-transcriptional modifications and degradation (Dissmeyer et al., 2019). Dysregulated proteostasis changes the protein interactome, which culminates in pathological changes in the cells, leading to diseases including cancer. Another layer of regulation is provided at the RNA level. The abundance of RNA and proteins in a cell is the outcome of the balance between their production and degradation, that are regulated by the gene expression control system (Jolma et al., 2020). The relationship between proteins and RNA, which dates back to the discovery of ribosomes, is strongly entwined and coordinated, and witnesses the ancient and dynamic interaction between ribosomal RNA (rRNA) and ribosomal proteins to regulate translation (Palade, 1955; Catalanotto et al., 2023). Post-transcriptional regulation of gene expression takes place at different levels: RNA splicing and editing, stability and decay, translocation and localization as well as translation, which are mostly under the control of RNA-binding proteins (RBPs). RNA-binding domains, with over 600 types identified up to now, such as the RNA recognition motif, K homology motif, zinc-finger domain or double-stranded RNA-binding motif, characterize the RBPs and mediate protein-protein interactions (Yang et al., 2021). By forming a multilayered ribonucleoprotein (RNP) complex, RBPs steer RNA molecules throughout their molecular journey, starting from “generation” (transcription), progressing through “development” (maturation), sustaining an “operational phase” (molecular function), and persisting until “termination” (turnover) (Smirnov, 2022). RBPs can interact with various RNA species including messenger RNA (mRNA), microRNA (miRNA), long non-coding RNA (lncRNA), and circular RNA (circRNA) (Dong et al., 2023). RBPs identify their RNA targets either through direct interaction with the RNA bases of an unfolded RNA chain or by attaching to folded RNA structures (Jolma et al., 2020).

Fine-tuning of the expression of RBPs is crucial for maintaining a balanced cellular homeostasis. Due to their important role in RNA metabolism and their versatility in inherited as well as acquired diseases, RBPs are considered key modulators of pathological processes. Continuously evolving technology in genomics and bioinformatics has led to the identification of novel RBPs. These advancements enable comprehensive, high-throughput analysis of numerous samples at the molecular level, facilitating the *in silico* exploration of their functions. A first catalog of more than 1,500 RBPs encoded by the human genome and accounting for 7.5% of the protein-coding genes was proposed (Uchida et al., 2019). To underline the importance of RBPs in the cell, Kechavarzi *et al.* demonstrated how RBPs were more significantly expressed in human healthy and tumor tissues with respect to non-RBPs and other regulatory factors, and exhibited higher fluctuations in expression levels, which could change their RNP composition, hence setting the ground for pathogenesis (Kechavarzi and Janga, 2014). To this regard, we and others have previously shown how too much or too little of an RBP can activate oncogenic pathways, albeit through different mechanisms, in colorectal cancer (CRC). In particular, over-expression of the RBP, Epithelial Splicing Regulatory protein 1 (ESRP1), which are the “splicing masterminds” of epithelial cells, led to the surge of a partial Epithelial-to-mesenchymal transition (EMT) status in CRC cells, as happens with the downregulation of its expression, seemingly through the context-dependent association of this RBP with different RNPs (Fagoonee et al., 2017; Ala et al., 2020; Manco et al., 2021; Advani et al., 2023). Of late, dysregulation of RBPs were shown to modulate the responsiveness of tumor cells to chemotherapeutic drugs by binding specific sequences in the 3′-UTRs of target mRNAs to enhance or hinder mRNA translation; forming complexes with other proteins, including RBPs, within RNPs; promoting the formation of new splice variants; and prompting nuclear/cytoplasmic translocation (Mir et al., 2022; Cen et al., 2023). For instance, the oncogenic RBP Musashi 2 (MSI2) was shown to be highly expressed in hepatocellular carcinoma, and was associated with cancer stem cell stemness and chemoresistance through the activation of LIN28A, another RBP (Fang et al., 2017). The complex interaction network between RBPs and the tumor-related RNA targets in hepatocarcinogenesis has been recently extensively described (Zhang et al., 2022). Thus, RBPs and their association with other regulatory proteins hold the potential to become therapeutic targets in cancer.

However, despite the master regulatory role of RBPs in crucial cellular and extracellular processes, deregulation in RBP expression or functionality in human pathologies remain hitherto understudied. RBP expression alterations with consequent aberrantly-composed RNPs may act as drivers of pathogenesis, and if detected early, can help control disease evolution (Glaß et al., 2020). Recently, RBPs signature has been explored as potential biomarker mainly in some types of human cancer. Abnormally expressed RBPs such as insulin-like growth factor 2 (IGF2) has been shown to play a crucial role in the occurrence and development of reproductive tract tumors by influencing oncogenic processes including apoptosis, proliferation, epithelial mesenchymal transition (EMT), invasion and metastasis and drug resistance, through binding to 5′ UTR of *IGF2* (Xu et al., 2022). Moreover, analysis of differentially expressed RBPs in cervical squamous cell carcinoma and endocervical adenocarcinoma (CEST) and paracancerous tissues generated a list of 10 genes (DDX26B, SNRPN, RBM38, HENMT1, RNASEH2A, LRRFIP1, GAPDH, AIMP2, ANGEL2 and PRPF40B) promising as an independent prognostic, immune therapy and chemotherapy resistance marker, and showed higher accuracy with respect to other clinical parameters such as age, stage and grade (Chen et al., 2024). A RBPs signature was also generated as potential biomarker for the diagnosis and prognosis of esophageal squamous cell carcinoma (ESCC) (Yang et al., 2021). TCGA database was screened for RBPs differentially expressed in tumors *versus* normal samples, and a set of 7 RBPs (CLK1, DDX39A, EEF2, ELAC1, NKRF, POP7 and SMN1), most of which were related to overall survival of ESCC individuals, were obtained as prognostic model. Of these, increased levels of CLK1 and decreased expression of POP7 in tumor tissues compared to controls could predict a worse prognosis of the ESCC patients. RBPs can also participate in mechanisms bridging inflammation and cancer. It was shown that RBPs play an important role in oxidative stress responses, inflammaging and senescence-associated secretory phenotype (SASP) that characterize lung cancer and chronic obstructive pulmonary disease (COPD) (Salvato et al., 2023). RBPs regulate SASP and activation of pro-inflammatory pathways which lead to the secretion and release of several pro-inflammatory mediators such as cytokines, chemokines and growth factors. For instance, the RBP HuR positively regulates mRNA stability and translation of several SASP mediator transcripts such as Transforming Growth Factor (TGF)-β, Matrix Metalloprotease (MMP)9, Interleukin (IL)-1α, Tumor Necrosis Factor (TNF)-α, all known to be involved in tumorigenic pathways (Salvato et al., 2023). On the other hand, other RBPs such as TTP and AUF-1 promote SASP factors mRNA (example, TNF-α, IL-6) destabilization. Inflammatory response of immune cells to tissue injury or to pathogenic infection is also regulated by RBPs. For example, the C-C motif chemokine ligand 2 (CCL2) 5′UTR is bound by the large ribosomal subunit protein L22 (RPL22, a canonical RBP) to regulate CCL2 expression in lipopolysaccharide-stimulated THP-1 macrophages (Das et al., 2020). RBPs can regulate several immunological processes, such as those involved in autoimmune pathogenesis and organ inflammation (Akira and Maeda, 2021; Liu and Cao, 2023). RBPs can in turn undergo post-transcriptional and post-translational modifications by pathogenic and inflammatory signals. This mutual regulation guarantees finely-tuned and context-dependent responses of immune cells, thus playing a role in maintaining an equilibrium between tolerance and immunity (Akira and Maeda, 2021; Liu and Cao, 2023).

### 1.2 RNA metabolism and RNA-binding proteins in biliary tract disorders

Few studies have analysed RBPs expression dysregulation in cholestatic diseases. RBPs are involved in several steps of bile acid metabolism, which is disrupted upon cholestasis, and is one of the very early events leading to chronic cholestatic liver diseases. The RBP, *Zfp36l1*, a target gene of the key regulator of bile acid homeostasis, Farnesoid X receptor (FXR), was found to post-transcriptionally modulate *Cyp7a1* mRNA stability and bile acid levels in rodents (Tarling et al., 2017). In particular, ZFP36L1 enhances, through recruitment of other proteins such as deadenylases, the degradation rate of *Cyp7a1* mRNA by binding to AU-rich elements in its 3′-UTR, hence leading to reduced bile acid synthesis. ZFP36L1 is a canonical RBP with several putative FXR response elements in the gene locus, and a functional FXRE in the promoter region (proximal) of the *Zfp36l1* gene (Tarling et al., 2017). The authors showed that the absence of ZFP36L1 altered bile acid metabolism, and consequently, impaired lipid absorption in mice, thus dampening the development of diet-induced obesity and steatosis. In another study, the lncRNA maternally expressed gene 3 (*MEG3*) was found to bind with the RBP polypyridine tract-binding protein 1 (PTBP1). This interaction led to the degradation of a PTBP1 mRNA target, small heterodimer partner (SHP), a critical regulator of bile acid synthesis, hence promoting cholestatic liver injury in rodents (Zhang et al., 2017). Importantly, *PTBP1* expression was significantly increased in the livers of patients with fibrosis and metabolic dysfunction-associated steatohepatitis (MASH, previously known as NASH) compared to normal or steatotic individuals. RBPs also regulate key processes during liver fibrogenesis, and are differentially expressed during the hepatic stellate cell (HSC) transdifferentiation into myofibroblasts. Wang *et al.* found that the canonical RBPs, Insulin-like growth factor 2 binding protein 3 (Igf2bp3), CUGBP Elav-like family member 2 (Celf2) and RNA Binding Motif Protein 47 (Rbm47) are differentially modulated and are involved in the conversion of HSC to myofibroblasts (Wang et al., 2018). Depletion of *Igf2bp3* in HSCs, for instance, reduced the proliferative capacity of these cells, and dampened the transcriptomic changes that occur during their conversion to the myofibrolastic cells. Albeit the composition of myofibroblast may vary according to pathogenesis of liver fibrosis, similar RBP-involving events may occur in the activation of portal fibroblasts leading to hepatobiliary fibrosis in chronic cholestatic diseases such as PSC, which warrants further investigation (Wu et al., 2021).

In the context of PSC and CCA, several studies have been performed to identify non-coding RNAs as novel biomarkers (Table 1) (Voigtländer et al., 2015; Bernuzzi et al., 2016). Some miRNAs (such as miR-1281, -126, −122, −26a, −30b) are very promising for distinguishing between PSC and CCA, and show high diagnostic specificity (Voigtländer et al., 2015). However, despite their role as master regulator in crucial cellular processes, the utility of RBPs as diagnostic or prognostic markers in clinical setting is largely undefined. The RBP transcripts and proteins are also one of the molecular links that can bridge the gap between PSC and CCA. Puthdee *et al.* showed that the overexpression of the RBP LIN28B could increase inflammatory cytokines (including IL-8, IL-6, VEGF, MCP1, TNF-α) release as well as resistance to chemotherapeutic drugs (Cisplatin, Gemcitabine, Etoposide) of cholangiocytes (MMNK-1 cells) through activation of the STAT3 signaling pathway, which could in part contribute to the initiation of CCA (Puthdee et al., 2021). Another study revealed the potential of the RBP Hu-antigen R (HuR) as prognostic marker of CCA patients’ outcome (Toyota et al., 2018). Cytoplasmic *HuR* expression was increased and predictive of poor disease-free survival and overall survival of subjects with surgically-resected CCA who underwent adjuvant gemcitabine-based chemotherapy. To our knowledge, there have been no studies directly examining the dysregulated expression of RBPs in PSC. However, comprehensive transcriptomic analyses of samples from PSC patients have revealed several RBPs that exhibit differential expression compared to controls (Ostrowski et al., 2019; Lapitz et al., 2020; Lei et al., 2022). We attempted herein to extract information from the transcriptomic repository regarding the potential significance of RBP transcripts that show dysregulated expression in PSC and CCA as biomarkers. One essential requisite to be defined as biomarker is the non-invasiveness of the material sampling procedure. Thus, we further our research into potential RBP transcripts as biomarkers for PSC and CCA, by performing a bioinformatics analysis to investigate whether RBPs could be found differentially present in liver tissue and body fluids (as well as in extracellular vesicles (EVs), which are considered as one of the optimal sources of biomarkers due to their biogenesis and the molecular information they carry from the source organs) (Ferro et al., 2024).

**TABLE 1:** Examples of differentially expressed Non-coding RNAs (ncRNAs) in PSC and CCA biofluids potentially useful as biomarkers.

Disease	Body fluid	ncRNA type	ncRNA	Expression	References
PSC	serum	miRNAs	miR-200c	-Down^a^	Bernuzzi et al. (2016)
			miR-1281, miR-126, miR-26a, miR-30b, miR122	-Up^a^ ^,^ ^b^	Voigtländer et al. (2015), Bernuzzi et al. (2016)
			**miR‐193b, miR‐122 and miR‐885‐5p**	-Up^a^	Bernuzzi et al. (2016)
	bile		miR-215, miR-194, miR-132, miR-412, miR-192, miR-1537, miR-640, miR-302b*, and miR-3189	-Up^b^	Voigtländer et al. (2015)
			miR-1537, miR-412, miR-640, miR-3189	Down^c^	Voigtländer et al. (2015)
CCA	serum		miR‐483‐5p, miR‐194	-Up^a^	Bernuzzi et al. (2016)
			miR‐222, miR‐483‐5p	-Up^b^	Bernuzzi et al. (2016)
			miR-1281, miR-126, miR-26a, miR-30b, miR122	-Up^a^ -Down^b^	Voigtländer et al. (2015)
			**miR‐193b, miR‐122 and miR‐885‐5p**	-Up^a^	Bernuzzi et al. (2016)
	bile		miR-1537, miR-412, miR-640, miR-3189	-Down^b^ -Down^c^	Voigtländer et al. (2015)

^a^ PSC or CCA vs. Controls.

^b^ PSC vs*.* CCA.

^c^ PSC or CCA vs. PSC/CCA; in **bold**: miRNAs upregulated in both PSC and CCA vs. controls.

That RBPs (as protein or RNA) can be released by cells and found in body fluids is not a new notion, but remains hitherto understudied. RBPs play a significant role in sorting non-coding RNAs (ncRNAs) into EV (Mateescu et al., 2017; Statello et al., 2018). These ncRNAs, transported by EV, contribute to the regulation of various aspects of tumor progression, including metastasis, angiogenesis, modulation of the tumor microenvironment, and resistance to drugs (Wang and Zhang, 2023). For instance, the RBP Heterogeneous nuclear ribonucleoprotein A1 (hnRNPA1), which regulates RNA metabolism, has been found to promote the packaging of miR-27b-3p into EV to facilitate CRC cells metastasis through the vasculature (Dou et al., 2021). One of the most studied RBPs, the Argonaute (AGO) family proteins, play crucial roles as effector complexes (RNA-induced silencing complex (RISC)/miRNP complex) of silencing mechanisms by siRNAs and miRNAs. AGO proteins are potential blood biomarkers with diagnostic and prognostic values (Geekiyanage et al., 2020). For instance, by dosing the level of miRNA-21 bound to AGO2 in plasma, Fuji *et al.* could distinguish CRC patients from subjects without the disease (Fuji et al., 2019). Interestingly, a specific subgroup of RNAs (including RBP transcripts) can exit cells and enter the extracellular environment through the formation of vesicles such as exosomes, and are promising as biomarkers for monitoring early pathological changes in the liver, such as during chronic cholestasis in mouse models and patients (Fagoonee et al., 2022; Povero et al., 2022).

Therefore, conducting a comprehensive search for secreted RBPs (RNA and protein) is essential for identifying disease biomarkers. This effort may contribute to establishing a panel of biomolecules capable of diagnosing PSC and predicting patient outcomes. In the present systematic review, we use a bioinformatics approach to extract information on RBPs and related transcripts (as identified in the RBP World database, which has expanded the number of proteins identifiable as RBPs also by their action through non-canonical sites), showing dysregulated RNA expression in body fluids, and discuss their potentiality as biomarkers that may allow early diagnosis of PSC and determine their prognosis to CCA. We also briefly discuss how their aberrant expression renders them an appealing therapeutic objective for intervention in an effort on improving the grim prognosis of CCA.

## 2 Methods

### 2.1 Search strategy

The Cancer Genome Atlas (TCGA) consortium was selected to explore the CCA (CHOL) dataset. Data have been downloaded from: https://tcga-xena-hub.s3.us-east-1.amazonaws.com/download/TCGA.CHOL.sampleMap%2FHiSeqV2.gz (Farshidfar et al., 2017). The Gene Expression Omnibus (GEO) database at the NCBI has been mined to find expression data for PSC by imposing the following selection criteria: *Primary Sclerosis Cholangitis* and *Homo sapiens*, filtering for “*Expression profiling by array*” (Barrett et al., 2013). Seven GEO series were identified: GSE159676, GSE144521, GSE130563, GSE119600, GSE84954, GSE11908 and GSE11907 (the latest being a superseries including GSE11908). Series not explicitly containing samples for PSC subjects were excluded. GEO series GSE144521, GSE159676 and GSE119600 were retained for the subsequent analyses.

### 2.2 Data sources

The RBP World (EuRBPDB2) database, http://research.gzsys.org.cn/eurbpdb2/index.html, was selected to obtain the list of RBP in Human, counting for 2,853 unique proteins and the list of canonical and non-canonical RBPs was downloaded from the website (Liao et al., 2020). Tissue (gall bladder and liver; cancer versus normal) expression of identified RBPs were verified using the following databases: http://gent2.appex.kr/gent2/ (Table 2) (Park et al., 2019).

**TABLE 2:** List of GEO Series associated in GENT2 database (used for exploring Gene Expression patterns across Normal and Tumor tissues) for both liver and gallbladder cancer and normal tissues.

Dataset_ID	Liver-cancer	Liver-normal
GSE11045		3
GSE13471		5
GSE15238		6
GSE18462	2	2
GSE19665	10	10
GSE2109	46	
GSE23343	1	6
GSE29722	10	10
GSE31177		1
GSE31193		3
GSE33006	3	3
GSE36076	10	
GSE38598		2
GSE38941		10
GSE40367	30	
GSE40873		49
GSE41804	20	20
GSE43346		1
GSE45436	93	41
GSE49515	10	
GSE58208	10	5
GSE6222	10	2
GSE62232	81	10
GSE63068		7
GSE6764	37	8
GSE68638	3	
GSE71856	1	
GSE7307		5
GSE75285	50	5
GSE9843	90	1
Total	517	215
Dataset_ID	Gallbladder-Cancer	Gallbladder-Normal
GSE34166	6	4
GSE40367	7	
GSE43346		1
Total	13	5

For each Series the number of samples is reported, for both cancer and normal condition, as well as the total number of samples analyzed. All samples are based on the Affymetrix Human Genome U133 Plus 2.0 Array.

### 2.3 Data extraction and analysis

The CHOL TCGA dataset, from the TCGA Bile Duct Cancer cohort, was composed of 36 patients and 9 normal adjacent tumor tissue. The gene expression profile was measured using the Illumina HiSeq 2000 RNA Sequencing platform. Normalized data were downloaded as in log2(x+1) transformed RSEM normalized count and the differential expression was analyzed using the EBSeq library in R (blockmodeling package) (Žiberna and Cugmas, 2023).

The GSE119600 dataset provided 45 PSC and 47 control samples from adult whole blood; the GSE159676 dataset was used to study fresh frozen tissue obtained from livers, 6 liver tissue healthy and 12 PSC; the GSE144521 procured samples from serum and urine extracellular vesicles (EV) for PSC (6 serum and 6 urine samples), CAA (12 serum and 23 urine samples) and healthy control (9 serum and 5 urine samples) (Ostrowski et al., 2019; Lapitz et al., 2020; Lei et al., 2022). For all the three dataset, GEO2R web tool was used to study the differential expression, with default parameters. Probeset-gene annotation was obtained directly from the GEO2R output, or from the annotation file for the specific platform used as present in the GEO page itself.

**FIGURE 1:**
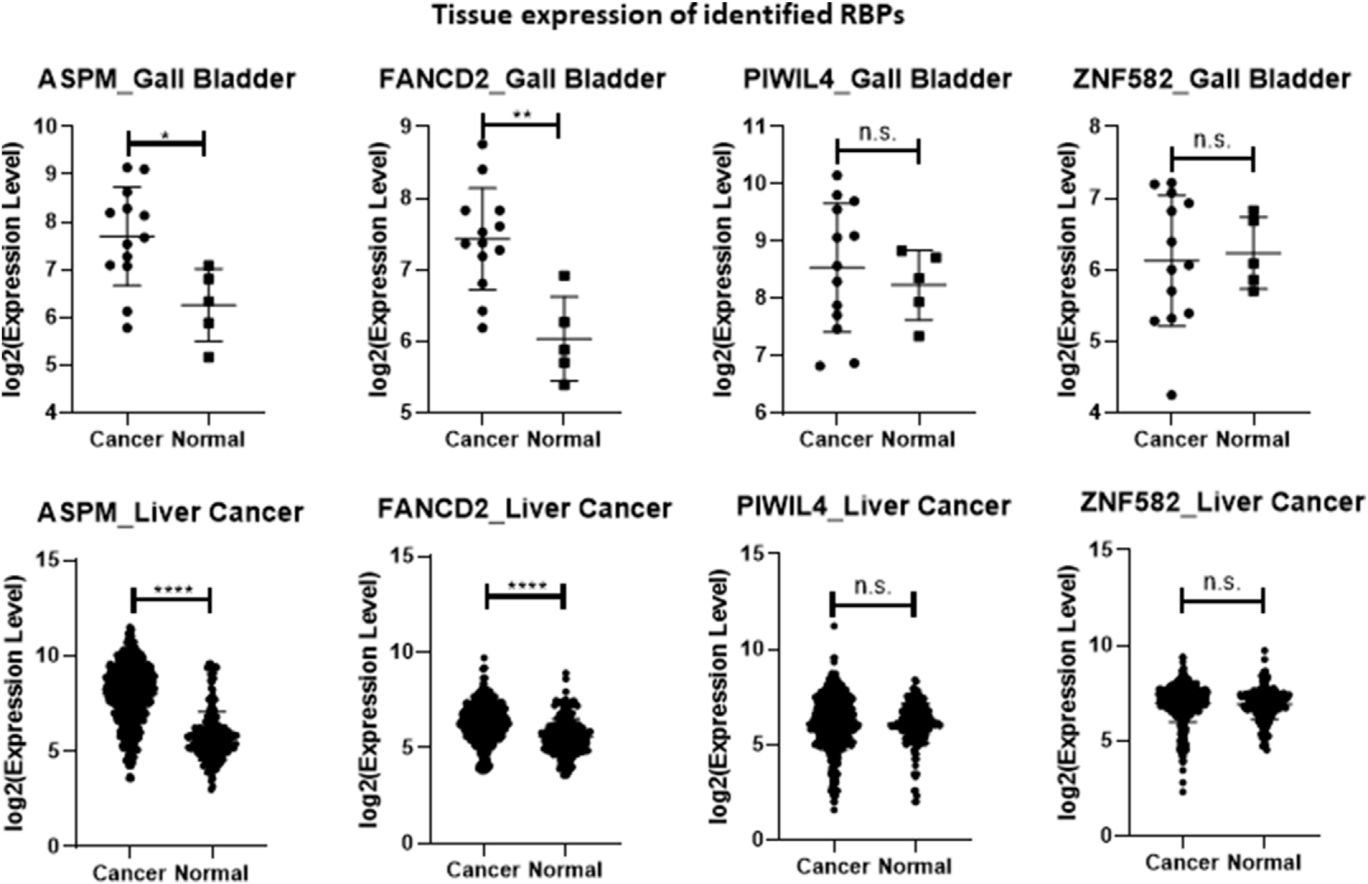
Plot of the four identified RBPs’ expression values according to the list of samples reported in Table 2 and obtained from GENT2 database (expression in normal *versus* cancer tissues). All values are downloaded into a log2 normalization. Statistical significance was assessed using a heteroscedastic two-tailed distribution Student’s t-test (*p < 0.05; **p < 0.01; ****p < 0.0001).

Analyses were conducted by using R (version 4.3.2) and RStudio (release 2023.06.1 Build 524). For tissue expression data from GENT2 (Figure 1), statistical significance was assessed using a heteroscedastic two-tailed distribution Student’s t-test (*p < 0.05; **p < 0.01; ****p < 0.0001).

The ability of the different transcripts to discriminate between normal and diseased conditions was evaluated through a Receiver Operating Curve (ROC) analysis in both datasets (GSE144521 and CHOL-TCGA). ROC analyses were performed in R with pROC library (version 1.18.5); in the text, AUC, specificity and sensitivity values are reported, while in the pictures, AUCs and their associated 95% Confidence Interval are reported.

## 3 Results

The results used here are entirely or partially based upon data generated by the TCGA Research Network: https://www.cancer.gov/tcga.

### 3.1 Database selection and characteristics

Data from CHOL TCGA and the different GEO datasets were analyzed to highlight differentially expressed genes and information on human RBP transcripts were subsequently extracted. In CHOL TCGA (Table 3), differentially expressed genes were selected with a posterior probability (PPDE) < 0.05 and the posterior fold change higher than 1.5 or lesser than 0.75 for up or downregulated, respectively. From this analysis, 2,192 upregulated and 1778 downregulated genes were identified in tumor context compared to normal samples, corresponding to a selection of 86 upregulated and 56 downregulated RBP transcripts (Table 3; Table 4), comprised of canonical and non-canonical RBPs.

**TABLE 3:** List of datasets used for the analysis.

Datasets	Status1 (N1)	Status2 (N2)	Sample origin	N. Up	N. Down	N. RBP up	N. RBP down
CHOL TCGA	CAA	NAT	Primary Cancer	2,192	1778	86	56
GSE144521	PSC	CTRL	Serum	500	381	65	56
GSE144521	PSC	CTRL	Urine	600	234	83	40
GSE144521	CAA	PSC	Serum	1,626	894	254	118
GSE144521	CAA	PSC	Urine	55	214	4	25
GSE159676	PSC	CTRL	Fresh frozen tissue	10	9	0	0
GSE119600	PSC	CTRL	Whole blood	29	70	2	29

Status1 = condition in the first group of comparison (CAA = Cholangiocarcinoma; PSC = Primary Sclerosing Cholangitis); Status2 = condition in the second group of comparison (NAT = normal adjacent tumor tissue; CTRL = normal and control condition, depending on the dataset; PSC = Primary Sclerosing Cholangitis); Sample Origin = specification of the biological material of the experiment; N. Up = number of the upregulated genes; N. Down = number of downregulated genes; N. RBP Up = number of RBPs in the list of upregulated genes; N. RBP Down = number of RBPs in the list of downregulated genes.

**TABLE 4:** Lists of Differentially Expressed human RNA-Binding Proteins (RBPs).

Dataset	DE RBPs	Gene symbols
TCGA CHOL	Upregulated	ADARB2 , AFF2 , ANLN , APOBEC1 , APOBEC2 , APOBEC4 , ARL14 , ASNS , ASPM , BARD1 , BLM , BOLL , CALML5 , CDKN2A , CELF4 , CENPF , CPSF4L , DDX4 , DLX2 , EDDM3A , EEF1A2 , EIF4E1B , ELAVL2 , ELAVL3 , ELAVL4 , ENOX1 , ERCC6L , ESRP1 , EXO1 , FABP5 , FANCD2 , HELLS , HIST1H1B , HIST1H1E , HIST1H4A , HIST1H4B , HIST1H4D , HIST1H4E , HIST1H4H , HIST1H4I , HIST4H4 , ITPR3 , KHDC1 , KIF14* , KIF23 , KIF2C , KIF4A , LIN28B , MAMSTR , MKI67 , MYEF2 , NCAPG , NCAPH , NEIL3 , NQO1 , PABPC1L2B , PFKP , PIWIL4 , PKP3 , POLQ , PRC1 , PRDM12 , PRDM5 , PTHLH , PURG , RAB25 , RAB36 , RAB6B , RAB9B , RAD51AP1 , RAD54L , RANBP17 , RASEF , RDM1 , RIMKLA , SMN1 , SRRM3 , TOP2A , TRIM71 , WDHD1 , ZBTB32 , ZC3HAV1L , ZFR2 , ZIC3 , ZNF233 , ZNF860
	Downregulated	A1CF , ACAT1 , ADAD2 , ALDOC , ANG , APOB , ARL4D , AS3MT , ASS1 , ASXL3 , AZGP1 , CALR3 , CAT , CLGN , CPEB3 , DDX25 , DDX3Y , DPPA5 , DPYD , EDDM3B , EIF1AY , ESR1 , GADD45G , HERC5 , HIST1H4F , HIST1H4J , HIST1H4L , HMGN5 , MBNL3 , METTL7A , METTL7B , MOV10L1 , MTHFD1 , NKIRAS1 , NXF3 , NYNRIN , PABPN1L , PAIP2B , PAPSS2 , PIWIL2 , PPARGC1B , PSAT1 , PSTK , PYGL , RASL11A , RBMXL2 , RCL1 , RPL3L , SALL1 , SLC16A1 , SRL , TDRD10 , ZBTB16 , ZFHX4 , ZFP1 , ZNF582
GSE119600	Upregulated	BASP1 , MBNL3
	Downregulated	ANP32A , BOLA2 , CAPZA1 , CBX3 , EIF1AX , EIF3M , G3BP1 , GIMAP1 , GIMAP2 , GIMAP7 , HIST1H4C , HSPE1 , MCTS1 , MRPL33 , RPL14 , RPL23 , RPL37A , RPL7 , RPL9 , RPLP1 , RPS21 , RPS27 , RPS28 , RPS29 , SNRPF , SUMO2 , TMA7 , UPF2 , YY1
GSE144521 Serum	Upregulated	ABCF2 , ANXA3 , APOBEC3F , APOBEC3G , ASPM , BCLAF1 , CAPZB , CCT3 , CD2BP2 , CLK3 , CNOT10 , CPEB2 , CPEB4 , CTR9 , DDX5 , EHD2 , ERN1 , GEMIN7 , GTPBP8 , H1F0 , HDLBP , HK1 , HSPA5 , ILF3 , INTS1 , KIDINS220 , LSM6 , MRPL30 , MRPS34 , MRTO4 , NAP1L4 , NDUFS1 , NKIRAS1 , NKTR , PICALM , PIWIL4 , POLR2K , POLR3B , POLR3H , PPIB , PPIG , PSMA1 , PSMC4 , QRSL1 , RAB23 , RNH1 , RTF1 , RUVBL1 , SAR1B , SEC61A1 , SHQ1 , SLC16A1 , SLTM , SRPK2 , SYNE1 , TCF7 , TFAM , TRMT61A , TTC14 , TXNDC5 , WDR3 , WDR61 , WRN , YRDC , ZNF579
	Downregulated	ABCF1 , ATXN2 , CNOT7 , DCTN1 , EIF2AK1 , EIF3M , EIF5A2 , ELAVL1 , FH , MBD1 , MRPS16 , MTRF1L , PATL1 , PDCD4 , PDCD7 , PNN , PNPT1 , POLR2J , PPA2 , PPIL3 , PRMT6 , PSMA6 , PSMC1 , RARS2 , ROCK2 , RPL19 , RPL23 , RPL27 , RPL41 , RPL7L1 , RPLP2 , RPS10 , RPS12 , RPS16 , RPS27 , RYBP , SMARCAL1 , SMN2 , TFIP11 , UPF3A , VCL , WTAP , ZBTB40 , ZBTB6 , ZC3HAV1L , ZNF2 , ZNF600 , ZNF652 , ZNF808 , ZNF860 , ZRANB1
GSE144521 Urine	Upregulated	ADARB1 , ANXA11 , AP1B1 , API5 , APOBEC3H , ARID4A , ASCC3 , BAG3 , CCDC86 , CCT5 , CNOT4 , CNOT7 , COPB2 , CYCS , DBN1 , DDX41 , DDX54 , DDX56 , DICER1 , DNMT3B , DRG1 , ERI3 , FAM129B , FANCD2 , FASN , FCF1 , FXR1 , G3BP2 , GSTCD , HIC2 , HNRNPC , IPO7 , KHNYN , KTN1 , LRBA , MDC1 , MEX3D , MRPL46 , MRPS11 , MRPS12 , MRPS16 , MRPS30 , NUFIP2 , OAS1 , OAS2 , PAPSS1 , PDCD4 , PDCD7 , PHF5A , PLD6 , PNPT1 , POLR1D , POP5 , POP7 , PRKDC , RBMS3 , RPL7L1 , RPS19BP1 , RPS27L , RPUSD2 , RRM1 , RRP7A , SDHA , SEC23IP , SMARCAL1 , SNRNP40 , SRP72 , STIP1 , SUPT4H1 , TARBP2 , TDRD1 , TFB1M , TFIP11 , TLR3 , TRIM33 , TTC14 , UBR4 , XPOT , YTHDC2 , ZC3H12A , ZMYND8 , ZNF385C , ZRANB2
	Downregulated	ACIN1 , ARHGEF2 , ATP1A1 , ATP6V1A , BAZ2A , BRCA1 , CCAR1 , CDC5L , CEPT1 , CSTF1 , CTU2 , FN1 , GLS , HNRNPA3 , HNRNPR , IMMT , LEMD3 , METTL2B , MRPL17 , MRPL43 , MYH9 , NCBP2 , NCL , NUDT21 , PHAX , PLCB3 , PPP1CB , PRPF8 , PSMD4 , RAVER1 , RBM33 , RPF2 , S100A4 , SPTBN1 , TXNL4A , UTP3 , WDR33 , ZMAT2 , ZNF582 , ZYX

Canonical RBPs are highlighted in red, non-canonical RBPs in blue, resulting from the downloaded version of the RBP World *Homo sapiens* database*;* in **bold**, genes common between tissue and body fluids, and showing similar expression pattern.

In the GEO datasets GSE159676 and in GSE119600, the differentially regulated genes had to show an absolute logFC higher than 0.5 and an adjusted *p-*value lesser than 0.05. Ten upregulated and 9 downregulated genes were found but none was a RBP in the first dataset; instead 29 up- and 70 downregulated transcripts were found in the second dataset, associated with 2 up and 29 down RBPs (Table 3; Table 4). However, the overlap of these RBPs, differentially expressed and with a similar trend with those found in CHOL TCGA dataset was empty.

In GEO dataset GSE144521, the comparisons between PSC and controls in both serum and urine samples requested an absolute logFC higher than 1 and a *p*-value lesser than 0.05, bringing to 500 up- and 381 downregulated genes in serum and 600 up- and 234 downregulated genes in urine samples. The RBPs found differentially expressed were 65 up and 56 down in serum comparison and 83 up and 40 down in urine one (Table 3; Table 4).

### 3.2 RBP transcripts differentially expressed in body fluids in PSC and CCA versus healthy controls

The comparison of the expression of these modulated RBPs with those of CHOL TCGA highlighted 4 differentially expressed transcripts in both PSC and CHOL *versus* normal conditions, also characterized by similar expression pattern in the different comparisons: Assembly factor for spindle microtubules (*ASPM*) and Piwi Like RNA-Mediated Gene Silencing 4 (*PIWIL4*) upregulated in serum and Fanconi anemia complementation group D2 (*FANCD2*) upregulated in urine and Zinc finger protein 582 (*ZNF582*) downregulated in urine (Table 4; Table 5).

**TABLE 5:** Overlap of differentially expressed RNA-Binding proteins.

DE RBPs overlap		*TCGA_CHOL*	
		Up (86)	Down (56)
*GSE144521 Serum*	Up (65)	**2**	2
	Down (56)	3	**0**
*GSE144521 Urine*	Up (83)	**1**	0
	Down (40)	0	**1**
*GSE119600*	Up (2)	**0**	1
	Down (29)	0	**0**
*GSE159676*	Up (0)	NA	NA
	Down (0)	NA	NA

Transcripts found with the same expression trend in both databases are highlighted in bold.

We further analyzed the behavior of these 4 transcripts in the comparison of body fluid samples from CAA and PSC, as provided in GSE144521. *ASPM*, *PIWIL4* and *ZNF582* were not found differentially expressed, while one isoform of *FANCD2* (linked to NM_033084.3) was upregulated in tumor with respect to PSC (Table 4; Table 5).

### 3.3 Cancer tissue expression of ASPM and FANCD2

In an effort to obtain information on the tissue expression of the four selected RBP-related transcripts, we interrogated GENT2, a platform for exploring gene expression patterns across tumor and normal tissues (Park et al., 2019). In general, *ASPM* and *FANCD2* exhibited higher expression in cancerous tissues. In particular, the expression of *ASPM* and *FANCD2* were significantly upregulated in gallbladder cancer and liver cancer tissue samples *versus* their respective controls (Figure 1; Table 2). However, the mechanism behind their increased expression under diseased condition (CCA *versus* PSC) is not known, and needs to be further investigated. Conversely, although *PIWIL4* displayed increased expression in serum samples from PSC and CCA patients compared to controls, it did not exhibit differential expression in hepatobiliary cancerous *versus* normal tissues. Moreover, *ZNF582* was downregulated in the urine of PSC patients but did not show alterations in expression levels between gallbladder cancer *versus* normal tissue or liver cancer and normal tissue (Figure 1; Table 2).

### 3.4 ROC analysis

**FIGURE 2:**
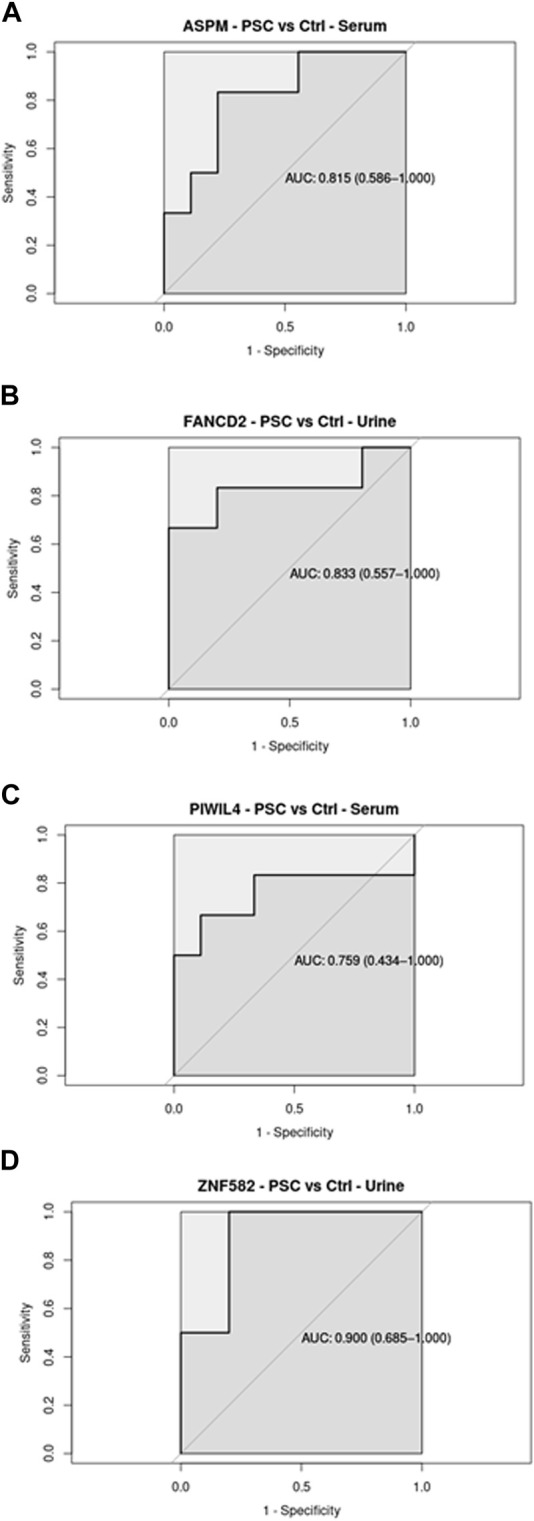
Receiver Operating Curve (ROC) analysis on Serum and Urine samples related to the four RBP transcripts in GSE144521 datasets, with their AUC values associated with the 95% Confidence Interval.

**FIGURE 3:**
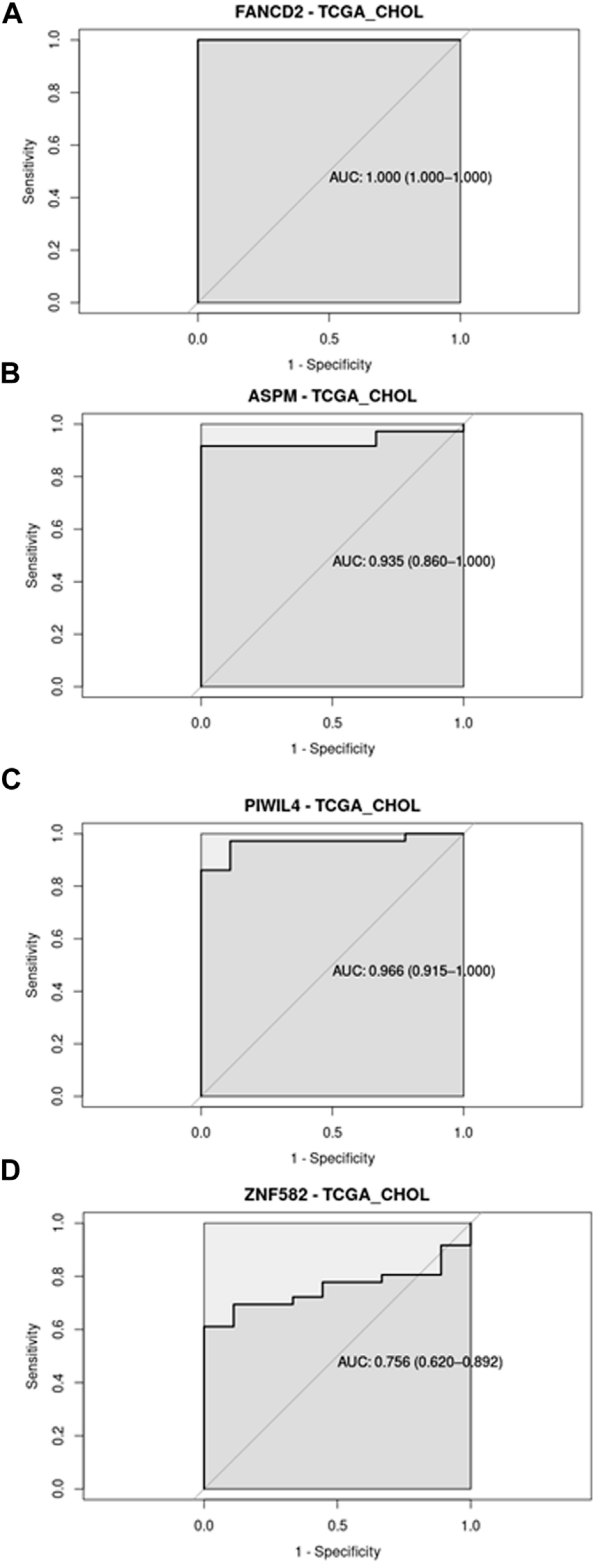
Receiver Operating Curve (ROC) analysis on CHOL-TCGA dataset related to the four RBP transcripts, with their AUC values associated with the 95% Confidence Interval.

Regarding the ROC analysis, the GSE144521 dataset showed promising results for ASPM (AUC = 81.5%; specificity = 77.78%; sensitivity = 83.33%) and PIWIL4 (AUC = 75.9%; specificity = 88.89%; sensitivity = 66.67%) in the Serum samples evaluation (Figures 2A, C), while for the urine samples, an encouraging ability to discriminate between conditions was observed with FANCD2 (AUC = 83.3%; specificity = 100%; sensitivity = 66.67%) and ZNF582 (AUC = 90.0%; specificity = 80%; sensitivity = 100%) transcripts (Figures 2B, D). On the other hand, the TCGA_CHOL samples gave an even stronger result with all the four transcripts showing a good ability to discriminate the tumor samples from the normal ones. The four transcripts are associated with these respective values: ASPM (AUC = 93.5%; specificity = 100%; sensitivity = 91.67%), PIWIL4 (AUC = 96.6%; specificity = 100%; sensitivity = 86.11%), FANCD2 (AUC = 100%; specificity = 100%; sensitivity = 100%), ZFN1582 (AUC = 75.6%; specificity = 100%; sensitivity = 61.11%) (Figures 3A–D). Overall, the ROC results were in line with the differential expression analysis, with the four differentially expressed RBP transcripts showing the best results in terms of AUC in the corresponding dataset.

## 4 Discussion

RBPs play a crucial role in a cell’s RNA metabolism. Any dysregulation in expression of RBPs can act as driver of cell transformation into a malignant phenotype and induce a cascade of molecular alterations which become uncontrollable over time. RBPs also participate in the regulation of therapy sensitivity and resistance (Cen et al., 2023). Thus, it is of prime importance to detect the dysregulation in the expression of key proteins, such as RBPs, very early before the onset of cancer, so that immediate clinical action can be taken. Liver biopsy is the gold standard for PSC staging and CCA detection, but is not exempt of complications (Fagoonee et al., 2022). Thus, analysis of body fluids in search of RBPs secreted as proteins or as transcripts enclosed in EVs, the expression of which mirrors those of the tissue of interest is an enthralling approach.

In the present systematic review, we provide an update on RBP transcripts that are involved in biliary tract disorders, with special focus on PSC and CCA, and we further our description by bioinformatically mining multiomics databases to extract unprecedented information on RBP transcripts that show promises as biomarkers for PSC and its progression into CCA. Most studies on biomarker search focus on upregulated biomolecules rather than downregulated ones, with the former being easier to validate clinically. Two out of the four RBP transcripts found upregulated in PSC and CCA in the present review, namely, *ASPM* and *FANCD2*, are indeed promising as potential biomarkers of PSC, as they show significant upregulation in expression also in PSC body fluids with respect to that of healthy controls. This could also be observed in the ROC analysis, which highlights the capacity of these biomolecules to distinguish between normal and disease conditions. While *ASPM* (a non-canonical RBP) expression remains constant between PSC and CCA, one isoform of *FANCD2* (a non-canonical RBP) shows an increase in expression from PSC to CCA in the body fluids, suggesting that *FANCD2* could be a candidate biomarker for the surge of CCA in PSC patients. Importantly, *ASPM* and *FANCD2* were significantly upregulated in hepatic and biliary cancer tissues (liver and gall bladder) with respect to controls, showing that there was positive association between tissue expression and secreted levels. Finding RBP transcripts in both tissue and body fluid samples, and especially showing similar differential expression patterns under normal *versus* pathological conditions, meets one of the criteria for a biomarker (Oloomi et al., 2020).

In order to understand whether the RBP transcripts identified in our study, as promising biomarkers for the identification of PSC patients *versus* healthy subjects, are also found in EV (exosomes), we interrogated the Vesiclepedia database (Chitti et al., 2024). Interestingly, *ASPM* was found in EV derived from serum, urine, and different types of cancer cells (Sarker et al., 2014); *FANCD2* in EV of serum, urine and CRC cells (Hong et al., 2009; Fraser et al., 2013); and *PIWIL4* (a canonical RBP) was also found in serum, urine and CRC cells (Hong et al., 2009; Musante et al., 2012; Fraser et al., 2013). The mRNA of these RBPs may be loaded onto EVs during their biogenesis, probably due to a zipcode-like sequence present in their 3′UTR (Chen et al., 2021). Interestingly, the expression of PIWIL4 (both mRNA and protein) as well as that of a transcription promoter-binding protein SUPT5H, were found significantly upregulated in intrahepatic CCA, and could be potentially used as prognostic markers for this cancer (Zou et al., 2021). On the other hand, *ZNF582* (a canonical RBP) does not localize to EV. This finding confirms previous reports on the presence of several RBP transcripts in EV (Statello et al., 2018).

ASPM, which is involved in mitotic spindle regulation and coordination of mitotic processes, has already been identified as a tumor marker in other settings. In an attempt to clarify the role of ASPM in tumor immunity and the prognosis of different cancers, Deng *et al.* analyzed the expression of ASPM in different tissues including kidney renal clear cell carcinoma and liver hepatocellular carcinoma (Deng et al., 2022). In these two types of cancers, higher expression of *ASPM* was evidenced in cancer tissues with respect to normal corresponding tissue, in late-stage cancers *versus* early-stage ones. Mechanistically, high *ASPM* expression correlated with poor overall patient survival and disease-specific survival, thus putting ASPM at the forefront as a prognostic biomarker. Importantly, ASPM expression was upregulated both at the mRNA and protein levels in liver cancer tissue, enhanced HCC cell proliferation and Epithelial-to-Mesenchymal Transition (EMT), as well as stimulated the Wnt––β‐catenin signaling by antagonizing the disheveled‐2 (Dvl2) degradation mediated by autophagy (Zhang et al., 2021). Recently, the role of ASPM in conferring a malignant phenotype and regulating cancer stemness was extensively reviewed (Tsai et al., 2023). *FANCD2*, which regulates ferroptosis, was also found overexpressed in Hepatitis B-related hepatocellular carcinoma (Tang et al., 2023). Its high expression was predictive of poor outcome of the disease, hence indicating FANCD2 as potential novel biomarker and immunotherapeutic target against Hepatitis B-related hepatocellular carcinoma. Several studies point out to an important role of FANCD2 in the initiation, development and progression of diverse tumors. To this regard, a recently published pan-cancer bioinformatics analysis, integrating several parameters such as gene expression and regulation, prognosis, and mutations across multiple cancer types, has shown that high FANCD2 expression was associated with poor prognosis in certain tumors (Zhao et al., 2024). On the whole, overall survival, disease-specific survival, or progression-free intervals was related to FANCD2 expression in certain cancerous tissues, including those of lung, breast, liver, and colon (Zhao et al., 2024). Thus, FANCD2 is promising as a diagnostic biomarker as well as a therapeutic target in multiple cancers. Another recent pan-cancer analysis highlights similar findings by showing that FANCD2 expression is augmented in most tumors present in the TCGA database, and further demonstrated that FANCD2 significantly enhances proliferation, migration and invasion capacity of lung adenocarcinoma cells (A549 and H1299 cells) through the regulation of cell cycle (Xie et al., 2024). FANCD2 can form RNPs with several proteins including the RBP heterogeneous nuclear ribonucleoprotein U (hnRNP U), as well as the ATP-dependent RNA helicases, DDX47, a member of the DEAD box protein family (Okamoto et al., 2019). FANCD2 has been shown to co-localize with R-loops in actively transcribed genomic regions, and the pairing with FANCI to form the I-D2 complex can directly bind RNA with a predilection for single-stranded RNA with G-rich sequence as well as R-loop structures with high affinity (Liang et al., 2019).

Targeting RBPs may be a promising tumor therapeutic strategy for PSC and CCA cancer patients. Although there is an increase in the development of new drugs targeting RBPs and associated factors, and new therapeutic strategies are being discovered, there are still many questions that need to be answered (Cen et al., 2023). These strategies may focus on the RBP itself, its RNA interaction, the up/downstream alterations to the proteome brought on by changes in RBP function, or any combination of these possibilities (Aguilar-Garrido et al., 2022). However, there are numerous challenges associated with the use of RBP inhibitors. For instance, many RBPs serve as pivotal regulators in cancer, acting as context-dependent oncogenes or tumor suppressors. Additionally, most RBPs have multiple cellular locations corresponding to their functions and exhibit promiscuity with numerous targets and functions. These factors can contribute to non-specificity, side effects, and toxicity of the potential RBP inhibitors. Therefore, it is imperative to comprehend the physiological implications of altered RBPs, the types of complexes they form, their dynamics, the role of post-translational modifications in altered RBPs, and the structural information required to target RBPs (isolated RBP versus RBP-RNA complex). To our knowledge, drugs targeting specifically ASPM and FANCD2 have not yet been developed, although there are indications on how to proceed to target ASPM and FANCD2 (targeting ASPM through the ASPM-TPX2-Cyclin B2 axis and FANCD2 through molecular inhibition of PI3K-AKT-mTOR, Ras-MAPK and CDK4) (Shen and Houghton, 2014; Pan et al., 2023).

## 5 Conclusion

Although there are some limitations to the current study, including the fact that the same patients were not longitudinally followed over time in the studies from which data were extracted, the two RBP-related transcripts identified in the present comprehensive review may develop into promising as biomarkers for the detection of PSC and predicting its outcome. These transcripts are overexpressed in hepatobiliary tissues of cancer patients as well as in body fluids of PSC and CCA subjects with respect to those of controls. Importantly, ASPM and FANCD2 can be vehiculated by EV in the body fluid, rendering these biomolecules even more attracting as biomarkers. This aspect should be investigated in more depth in future studies. There are also challenges in analysing RBP transcripts. Although post-transcriptional modulation of gene expression is continuous and dynamic, whether alterations in RBP expression are enough to reflect the changes in function is still unclear. Moreover, EV-sorted transcripts enrichment may not always correlate with tissue expression of these genes. Thus, it is important to carry out analysis of patient-derived tissue and circulating EVs in parallel. Data evincing from this systematic review sets the ground for further experimental research in this field.

## References

[B1] Advani R. Luzzi S. Scott E. Dalgliesh C. Weischenfeldt J. Munkley J. (2023). Epithelial specific splicing regulator proteins as emerging oncogenes in aggressive prostate cancer. Oncogene 42 (43), 3161–3168. 10.1038/s41388-023-02838-9 37752235 PMC10589096

[B2] Aguilar-Garrido P. Otero-Sobrino Á. Navarro-Aguadero M. Velasco-Estévez M. Gallardo M. (2022). The role of RNA-binding proteins in hematological malignancies. Int. J. Mol. Sci. 23 (17), 9552. 10.3390/ijms23179552 36076951 PMC9455611

[B3] Akira S. Maeda K. (2021). Control of RNA stability in immunity. Annu. Rev. Immunol. 39, 481–509. 10.1146/annurev-immunol-101819-075147 33577347

[B4] Ala U. Manco M. Mandili G. Tolosano E. Novelli F. Provero P. (2020). Proteomics-based evidence for a pro-oncogenic role of ESRP1 in human colorectal cancer cells. Int. J. Mol. Sci. 21 (2), 575. 10.3390/ijms21020575 31963158 PMC7014300

[B5] Baria K. De Toni E. Yu B. Jiang Z. Kabadi S. Malvezzi M. (2022). Worldwide incidence and mortality of biliary tract cancer. Gastro Hep Adv. 1, 618–626. 10.1016/j.gastha.2022.04.007 PMC11307584 39132071

[B6] Barrett T. Wilhite S. E. Ledoux P. Evangelista C. Kim I. F. Tomashevsky M. (2013). NCBI GEO: archive for functional genomics data sets--update. Nucleic Acids Res. 41, D991–D995. 10.1093/nar/gks1193 23193258 PMC3531084

[B7] Bergquist A. Ekbom A. Olsson R. Kornfeldt D. Lööf L. Danielsson A. (2002). Hepatic and extrahepatic malignancies in primary sclerosing cholangitis. J. Hepatol. 36 (3), 321–327. 10.1016/s0168-8278(01)00288-4 11867174

[B8] Bernuzzi F. Marabita F. Lleo A. Carbone M. Mirolo M. Marzioni M. (2016). Serum microRNAs as novel biomarkers for primary sclerosing cholangitis and cholangiocarcinoma. Clin. Exp. Immunol. 185 (1), 61–71. 10.1111/cei.12776 26864161 PMC4908296

[B9] Catalanotto C. Barbato C. Cogoni C. Benelli D. (2023). The RNA-binding function of ribosomal proteins and ribosome biogenesis factors in human health and disease. Biomedicines 11 (11), 2969. 10.3390/biomedicines11112969 38001969 PMC10669870

[B10] Catanzaro E. Gringeri E. Burra P. Gambato M. (2023). Primary sclerosing cholangitis-associated cholangiocarcinoma: from pathogenesis to diagnostic and surveillance strategies. Cancers (Basel) 15 (20), 4947. 10.3390/cancers15204947 37894314 PMC10604939

[B11] Cen Y. Chen L. Liu Z. Lin Q. Fang X. Yao H. (2023). Novel roles of RNA-binding proteins in drug resistance of breast cancer: from molecular biology to targeting therapeutics. Cell Death Discov. 9 (1), 52. 10.1038/s41420-023-01352-x 36759501 PMC9911762

[B12] Chen X. Dong X. Li H. Wu T. Liu H. Wu J. (2024). RNA-binding proteins signature is a favorable biomarker of prognosis, immunotherapy and chemotherapy response for cervical cancer. Cancer Cell Int. 24 (1), 80. 10.1186/s12935-024-03257-w 38383371 PMC10882920

[B13] Chen Y. Zhao Y. Yin Y. Jia X. Mao L. (2021). Mechanism of cargo sorting into small extracellular vesicles. Bioengineered 12 (1), 8186–8201. 10.1080/21655979.2021.1977767 34661500 PMC8806638

[B14] Chitti S. V. Gummadi S. Kang T. Shahi S. Marzan A. L. Nedeva C. (2024). Vesiclepedia 2024: an extracellular vesicles and extracellular particles repository. Nucleic Acids Res. 52 (1), D1694–D1698. 10.1093/nar/gkad1007 37953359 PMC10767981

[B15] Das A. S. Basu A. Kumar R. Borah P. K. Bakshi S. Sharma M. (2020). Post-transcriptional regulation of C-C motif chemokine ligand 2 expression by ribosomal protein L22 during LPS-mediated inflammation. FEBS J. 287 (17), 3794–3813. 10.1111/febs.15362 32383535

[B16] Deng T. Liu Y. Zhuang J. Tang Y. Huo Q. (2022). ASPM is a prognostic biomarker and correlates with immune infiltration in kidney renal clear cell carcinoma and liver hepatocellular carcinoma. Front. Oncol. 12, 632042. 10.3389/fonc.2022.632042 35515103 PMC9065448

[B17] Dissmeyer N. Coux O. Rodriguez M. S. Barrio R. (2019). PROTEOSTASIS: a European network to break barriers and integrate science on protein homeostasis. Trends Biochem. Sci. 44 (5), 383–387. 10.1016/j.tibs.2019.01.007 30777377

[B18] Dong P. Taheri M. Wang F. (2023). Editorial: interplay between RNA-binding proteins and non-coding RNAs in tumor therapeutic resistance. Front. Oncol. 13, 1201122. 10.3389/fonc.2023.1201122 37152004 PMC10159050

[B19] Dou R. Liu K. Yang C. Zheng J. Shi D. Lin X. (2021). EMT-cancer cells-derived exosomal miR-27b-3p promotes circulating tumour cells-mediated metastasis by modulating vascular permeability in colorectal cancer. Clin. Transl. Med. 11 (12), e595. 10.1002/ctm2.595 34936736 PMC8694332

[B20] Fagoonee S. Arigoni M. Manco M. Olivero M. Bizzaro F. Magagnotti C. (2022). Circulating extracellular vesicles contain liver-derived RNA species as indicators of severe cholestasis-induced early liver fibrosis in mice. Antioxid. Redox Signal 36, 480–504. 10.1089/ars.2021.0023 34779230 PMC8978575

[B21] Fagoonee S. Picco G. Orso F. Arrigoni A. Longo D. L. Forni M. (2017). The RNA-binding protein ESRP1 promotes human colorectal cancer progression. Oncotarget 8 (6), 10007–10024. 10.18632/oncotarget.14318 28052020 PMC5354637

[B22] Fang T. Lv H. Wu F. Wang C. Li T. Lv G. (2017). Musashi 2 contributes to the stemness and chemoresistance of liver cancer stem cells via LIN28A activation. Cancer Lett. 384, 50–59. 10.1016/j.canlet.2016.10.007 27721018

[B23] Farshidfar F. Zheng S. Gingras M. C. Newton Y. Shih J. Robertson A. G. (2017). Integrative genomic analysis of cholangiocarcinoma identifies distinct IDH-mutant molecular profiles. Cell Rep. 18 (11), 2780–2794. 10.1016/j.celrep.2017.02.033 28297679 PMC5493145

[B24] Ferro A. Saccu G. Mattivi S. Gaido A. Herrera Sanchez M. B. Haque S. (2024). Extracellular vesicles as delivery vehicles for non-coding RNAs: potential biomarkers for chronic liver diseases. Biomolecules 14, 277. 10.3390/biom14030277 38540698 PMC10967855

[B25] Fossdal G. Mjelle A. B. Wiencke K. Bjørk I. Gilja O. H. Folseraas T. (2021). Fluctuating biomarkers in primary sclerosing cholangitis: a longitudinal comparison of alkaline phosphatase, liver stiffness, and ELF. JHEP Rep. 3 (5), 100328. 10.1016/j.jhepr.2021.100328 34485881 PMC8403583

[B26] Fraser K. B. Moehle M. S. Daher J. P. Webber P. J. Williams J. Y. Stewart C. A. (2013). LRRK2 secretion in exosomes is regulated by 14-3-3. Hum. Mol. Genet. 22 (24), 4988–5000. 10.1093/hmg/ddt346 23886663 PMC3836478

[B27] Fuji T. Umeda Y. Nyuya A. Taniguchi F. Kawai T. Yasui K. (2019). Detection of circulating microRNAs with Ago2 complexes to monitor the tumor dynamics of colorectal cancer patients during chemotherapy. Int. J. Cancer 144 (9), 2169–2180. 10.1002/ijc.31960 30381824 PMC6590166

[B28] Geekiyanage H. Rayatpisheh S. Wohlschlegel J. A. Brown R. Ambros V. (2020). Extracellular microRNAs in human circulation are associated with miRISC complexes that are accessible to anti-AGO2 antibody and can bind target mimic oligonucleotides. Proc. Natl. Acad. Sci. U. S. A. 117 (39), 24213–24223. 10.1073/pnas.2008323117 32929008 PMC7533700

[B29] Glaß M. Michl P. Hüttelmaier A. S. (2020). RNA binding proteins as drivers and therapeutic target candidates in pancreatic ductal adenocarcinoma. Int. J. Mol. Sci. 21 (11), 4190. 10.3390/ijms21114190 32545414 PMC7312628

[B30] Hong B. S. Cho J. H. Kim H. Choi E. J. Rho S. Kim J. (2009). Colorectal cancer cell-derived microvesicles are enriched in cell cycle-related mRNAs that promote proliferation of endothelial cells. BMC Genomics 10, 556. 10.1186/1471-2164-10-556 19930720 PMC2788585

[B31] Jolma A. Zhang J. Mondragón E. Morgunova E. Kivioja T. Laverty K. U. (2020). Binding specificities of human RNA-binding proteins toward structured and linear RNA sequences. Genome Res. 30 (7), 962–973. 10.1101/gr.258848.119 32703884 PMC7397871

[B32] Kechavarzi B. Janga S. C. (2014). Dissecting the expression landscape of RNA-binding proteins in human cancers. Genome Biol. 15 (1), R14. 10.1186/gb-2014-15-1-r14 24410894 PMC4053825

[B33] Lapitz A. Arbelaiz A. O'Rourke C. J. Lavin J. L. Casta A. Ibarra C. (2020). Patients with cholangiocarcinoma present specific RNA profiles in serum and urine extracellular vesicles mirroring the tumor expression: novel liquid biopsy biomarkers for disease diagnosis. Cells 9 (3), 721. 10.3390/cells9030721 32183400 PMC7140677

[B34] Lei L. Bruneau A. El Mourabit H. Guégan J. Folseraas T. Lemoinne S. (2022). Portal fibroblasts with mesenchymal stem cell features form a reservoir of proliferative myofibroblasts in liver fibrosis. Hepatology 76 (5), 1360–1375. 10.1002/hep.32456 35278227

[B35] Liang Z. Liang F. Teng Y. Chen X. Liu J. Longerich S. (2019). Binding of FANCI-FANCD2 complex to RNA and R-loops stimulates robust FANCD2 monoubiquitination. Cell Rep. 26 (3), 564–572. 10.1016/j.celrep.2018.12.084 30650351 PMC6350941

[B36] Liao J. Y. Yang B. Zhang Y. C. Wang X. J. Ye Y. Peng J. W. (2020). EuRBPDB: a comprehensive resource for annotation, functional and oncological investigation of eukaryotic RNA binding proteins (RBPs). Nucleic Acids Res. 48 (1), D307–D313. 10.1093/nar/gkz823 31598693 PMC6943034

[B37] Liu J. Cao X. (2023). RBP-RNA interactions in the control of autoimmunity and autoinflammation. Cell Res. 33 (2), 97–115. 10.1038/s41422-022-00752-5 36599968 PMC9892603

[B38] Manco M. Ala U. Cantarella D. Tolosano E. Medico E. Altruda F. (2021). The RNA-binding protein ESRP1 modulates the expression of RAC1b in colorectal cancer cells. Cancers (Basel) 13 (16), 4092. 10.3390/cancers13164092 34439247 PMC8392041

[B39] Mateescu B. Kowal E. J. van Balkom B. W. Bartel S. Bhattacharyya S. N. Buzás E. I. (2017). Obstacles and opportunities in the functional analysis of extracellular vesicle RNA - an ISEV position paper. J. Extracell. Vesicles 6 (1), 1286095. 10.1080/20013078.2017.1286095 28326170 PMC5345583

[B40] Mir C. Garcia-Mayea Y. Lleonart M. E. (2022). Targeting the "undruggable": RNA-binding proteins in the spotlight in cancer therapy. Semin. Cancer Biol. 86 (3), 69–83. 10.1016/j.semcancer.2022.06.008 35772609

[B41] Mulinacci G. Cristoferi L. Palermo A. Luca M. Gerussi A. Invernizzi P. (2023). Risk stratification in primary sclerosing cholangitis. Minerva Gastroenterol. (Torino) 69 (1), 84–94. 10.23736/S2724-5985.20.02821-4 33300753

[B42] Musante L. Saraswat M. Duriez E. Byrne B. Ravidà A. Domon B. (2012). Biochemical and physical characterisation of urinary nanovesicles following CHAPS treatment. PLoS One 7 (7), e37279. 10.1371/journal.pone.0037279 22808001 PMC3395701

[B43] Okamoto Y. Abe M. Itaya A. Tomida J. Ishiai M. Takaori-Kondo A. (2019). FANCD2 protects genome stability by recruiting RNA processing enzymes to resolve R-loops during mild replication stress. FEBS J. 286 (1), 139–150. 10.1111/febs.14700 30431240

[B44] Oloomi M. Moazzezy N. Bouzari S. (2020). Comparing blood versus tissue-based biomarkers expression in breast cancer patients. Heliyon 6 (4), e03728. 10.1016/j.heliyon.2020.e03728 32274439 PMC7132155

[B45] Ostrowski J. Goryca K. Lazowska I. Rogowska A. Paziewska A. Dabrowska M. (2019). Common functional alterations identified in blood transcriptome of autoimmune cholestatic liver and inflammatory bowel diseases. Sci. Rep. 9 (1), 7190. 10.1038/s41598-019-43699-1 31076612 PMC6510750

[B46] Palade G. E. (1955). A small particulate component of the cytoplasm. J. Biophys. Biochem. Cytol. 1 (1), 59–68. 10.1083/jcb.1.1.59 14381428 PMC2223592

[B47] Pan H.-W. Lin Z.-Z. Huang G.-J. (2023). Abstract 1421: targeting ASPM suppresses the tumorigenicity of human hepatocellular carcinoma cells via ASPM-TPX2 axis disruption and result in increased chromosome segregation error. Cancer Res. 83, 1421. 10.1158/1538-7445.AM2023-1421

[B48] Park S. J. Yoon B. H. Kim S. K. Kim S. Y. (2019). GENT2: an updated gene expression database for normal and tumor tissues. BMC Med. Genomics 12 (5), 101. 10.1186/s12920-019-0514-7 31296229 PMC6624177

[B49] Povero D. Tameda M. Eguchi A. Ren W. Kim J. Myers R. (2022). Protein and miRNA profile of circulating extracellular vesicles in patients with primary sclerosing cholangitis. Sci. Rep. 12 (1), 3027. 10.1038/s41598-022-06809-0 35194091 PMC8863778

[B50] Puthdee N. Khramchantuk S. Nuwongsri P. (2021). LIN28B enhanced STAT3 signaling regulates inflammatory response and chemotherapeutic resistance in cholangiocytes. Asian Pac J. Cancer Prev. 22 (11), 3671–3678. 10.31557/APJCP.2021.22.11.3671 34837926 PMC9068185

[B51] Salvato I. Ricciardi L. Nucera F. Nigro A. Dal Col J. Monaco F. (2023). RNA-binding proteins as a molecular link between COPD and lung cancer. COPD 20 (1), 18–30. 10.1080/15412555.2022.2107500 36655862

[B52] Sarker S. Scholz-Romero K. Perez A. Illanes S. E. Mitchell M. D. Rice G. E. (2014). Placenta-derived exosomes continuously increase in maternal circulation over the first trimester of pregnancy. J. Transl. Med. 12, 204. 10.1186/1479-5876-12-204 25104112 PMC4283151

[B53] Shen C. Houghton P. J. (2014). Targeting FANCD2 for therapy sensitization. Oncotarget 5 (11), 3426–3427. 10.18632/oncotarget.2070 24913333 PMC4116492

[B54] Smirnov A. (2022). Research progress in RNA-binding proteins. Int. J. Mol. Sci. 24 (1), 58. 10.3390/ijms24010058 36613501 PMC9820217

[B55] Statello L. Maugeri M. Garre E. Nawaz M. Wahlgren J. Papadimitriou A. (2018). Identification of RNA-binding proteins in exosomes capable of interacting with different types of RNA: RBP-facilitated transport of RNAs into exosomes. PLoS One 13 (4), e0195969. 10.1371/journal.pone.0195969 29689087 PMC5918169

[B56] Tang X. Luo B. Huang S. Jiang J. Chen Y. Ren W. (2023). FANCD2 as a novel prognostic biomarker correlated with immune and drug therapy in Hepatitis B-related hepatocellular carcinoma. Eur. J. Med. Res. 28 (1), 419. 10.1186/s40001-023-01411-0 37821996 PMC10566141

[B57] Tarling E. J. Clifford B. L. Cheng J. Morand P. Cheng A. Lester E. (2017). RNA-binding protein ZFP36L1 maintains posttranscriptional regulation of bile acid metabolism. J. Clin. Invest. 127 (10), 3741–3754. 10.1172/JCI94029 28891815 PMC5617661

[B58] Tornai D. Ven P. L. Lakatos P. L. Papp M. (2022). Serological biomarkers for management of primary sclerosing cholangitis. World J. Gastroenterol. 28 (21), 2291–2301. 10.3748/wjg.v28.i21.2291 35800183 PMC9185217

[B59] Toyota K. Murakami Y. Kondo N. Uemura K. Nakagawa N. Takahashi S. (2018). Cytoplasmic hu-antigen R (HuR) expression is associated with poor survival in patients with surgically resected cholangiocarcinoma treated with adjuvant gemcitabine-based chemotherapy. Ann. Surg. Oncol. 25 (5), 1202–1210. 10.1245/s10434-018-6392-y 29492748

[B60] Tsai K. K. Bae B. I. Hsu C. C. Cheng L. H. Shaked Y. (2023). Oncogenic ASPM is a regulatory hub of developmental and stemness signaling in cancers. Cancer Res. 83 (18), 2993–3000. 10.1158/0008-5472.CAN-23-0158 37384617 PMC10502471

[B61] Uchida Y. Chiba T. Kurimoto R. Asahara H. (2019). Post-transcriptional regulation of inflammation by RNA-binding proteins via cis-elements of mRNAs. J. Biochem. 166 (5), 375–382. 10.1093/jb/mvz067 31511872 PMC6804641

[B62] Villard C. Friis-Liby I. Rorsman F. Said K. Warnqvist A. Cornillet M. (2023). Prospective surveillance for cholangiocarcinoma in unselected individuals with primary sclerosing cholangitis. J. Hepatol. 78 (3), 604–613. 10.1016/j.jhep.2022.11.011 36410555

[B63] Voigtländer T. Gupta S. K. Thum S. Fendrich J. Manns M. P. Lankisch T. O. (2015). MicroRNAs in serum and bile of patients with primary sclerosing cholangitis and/or cholangiocarcinoma. PLoS One 10 (10), e0139305. 10.1371/journal.pone.0139305 26431155 PMC4591993

[B64] Wang S. Jung Y. Hyun J. Friedersdorf M. Oh S. H. Kim J. (2018). RNA binding proteins control transdifferentiation of hepatic stellate cells into myofibroblasts. Cell Physiol. Biochem. 48 (3), 1215–1229. 10.1159/000491987 30045014

[B65] WangT.ZhangH. (2023). Exploring the roles and molecular mechanisms of RNA binding proteins in the sorting of noncoding RNAs into exosomes during tumor progression. J. Adv. Res. 2023, 29. 10.1016/j.jare.2023.11.029 38030125

[B66] Wu H. Chen C. Ziani S. Nelson L. J. Ávila M. A. Nevzorova Y. A. (2021). Fibrotic events in the progression of cholestatic liver disease. Cells 10 (5), 1107. 10.3390/cells10051107 34062960 PMC8147992

[B67] Xie X. Zhao Y. Du F. Cai B. Fang Z. Liu Y. (2024). Pan-cancer analysis of the tumorigenic role of Fanconi anemia complementation group D2 (FANCD2) in human tumors. Genomics 116 (1), 110762. 10.1016/j.ygeno.2023.110762 38104669

[B68] Xu X. Shen H. R. Zhang J. R. Li X. L. (2022). The role of insulin-like growth factor 2 mRNA binding proteins in female reproductive pathophysiology. Reprod. Biol. Endocrinol. 20 (1), 89. 10.1186/s12958-022-00960-z 35706003 PMC9199150

[B69] Yang X. Han B. He Z. Zhang Y. Lin K. Su H. (2021). RNA-binding proteins CLK1 and POP7 as biomarkers for diagnosis and prognosis of esophageal squamous cell carcinoma. Front. Cell Dev. Biol. 9, 715027. 10.3389/fcell.2021.715027 34568328 PMC8458940

[B70] Zhang H. Yang X. Zhu L. Li Z. Zuo P. Wang P. (2021). ASPM promotes hepatocellular carcinoma progression by activating Wnt/β-catenin signaling through antagonizing autophagy-mediated Dvl2 degradation. FEBS Open Bio 11 (10), 2784–2799. 10.1002/2211-5463.13278 PMC8487047 34428354

[B71] Zhang K. Barry A. E. Lamm R. Patel K. Schafer M. Dang H. (2022). The role of RNA binding proteins in hepatocellular carcinoma. Adv. Drug Deliv. Rev. 182, 114114. 10.1016/j.addr.2022.114114 35063534

[B72] Zhang L. Yang Z. Trottier J. Barbier O. Wang L. (2017). Long noncoding RNA MEG3 induces cholestatic liver injury by interaction with PTBP1 to facilitate shp mRNA decay. Hepatology 65 (2), 604–615. 10.1002/hep.28882 27770549 PMC5258819

[B73] Zhao Z. Wang R. Song J. Ma F. Pan H. Gao C. (2024). Pancancer analysis of the prognostic and immunological role of FANCD2: a potential target for carcinogenesis and survival. BMC Med. Genomics 17 (1), 69. 10.1186/s12920-024-01836-4 38443946 PMC10916239

[B74] Žiberna A. Cugmas M. (2023) Generalized and classical blockmodeling of valued networks. R package version 1.1.5.

[B75] Zou W. Wang Z. Zhang X. Xu S. Wang F. Li L. (2021). PIWIL4 and SUPT5H combine to predict prognosis and immune landscape in intrahepatic cholangiocarcinoma. Cancer Cell Int. 21 (1), 657. 10.1186/s12935-021-02310-2 34876138 PMC8649993

